# Evaluating the efficacy of probiotics and ascorbic acid as anti-stress agents against heat stress in broiler chickens

**DOI:** 10.3389/fvets.2024.1482134

**Published:** 2024-10-22

**Authors:** Victory Osirimade Sumanu, Vinny Naidoo, Marinda Catharina Oosthuizen, Joseph Panashe Chamunorwa

**Affiliations:** ^1^Department of Anatomy and Physiology, Faculty of Veterinary Science, University of Pretoria, Pretoria, South Africa; ^2^Department of Paraclinical Sciences, Faculty of Veterinary Science, University of Pretoria, Pretoria, South Africa; ^3^Department of Veterinary Tropical Diseases, Faculty of Veterinary Science, University of Pretoria, Pretoria, South Africa

**Keywords:** heat stress, cloacal temperature, body surface temperature, probiotic, ascorbic acid, thermoregulation

## Abstract

Heat stress poses a substantial challenge to poultry production worldwide, highlighting the urgent need for effective management strategies. This study investigated the efficacy of probiotics (*Saccharomyces cerevisiae*) and ascorbic acid as antistress agents using cloacal and body surface temperatures (CT and BST) as heat stress biomarkers in broiler chickens. A total of 56 broiler chicks were used for the experiment and were divided into four distinct groups: control, probiotics (1 g/kg of feed), ascorbic acid (200 mg/kg of feed) and the combination of probiotics and ascorbic acid (1 g/kg and 200 mg/kg of feed, respectively). The study lasted 35 days; measurements were taken for ambient temperature (AT), CT, and BST. The ambient temperature in the pens consistently exceeded the thermoneutral zone (TNZ) established for broiler chickens. The CT values for broiler chickens in the probiotic group were significantly lower (*p* < 0.05) compared to the control group. Additionally, the BST values in the probiotic and probiotic + ascorbic acid groups were significantly lower (*p* < 0.05) than those in the control group. The findings suggest that incorporating probiotics, with or without ascorbic acid, can effectively reduce CT and BST values in broiler chickens thereby, enhancing thermoregulation when compared to the control group. This implies that using probiotics in poultry diets may enhance health and growth performance, potentially leading to better feed efficiency and reduced reliance on antibiotics. Implementing these dietary strategies could improve the productivity and welfare of broiler chickens in commercial settings.

## 1 Introduction

Agricultural systems around the globe are increasingly facing negative consequences due to climate change. This is manifested in rising global temperatures and changing weather patterns (1). According to Sundstrom et al. (2), heightened temperatures, unpredictable rainfall, prolonged droughts, and more frequent extreme weather events pose significant threats to food production and security. These alterations disrupt the delicate balance of ecosystems, affecting the growth, development, and productivity of crops and livestock (3). In particular, high ambient temperatures during the summer months often lead to heat stress in broiler chickens (4, 5). This issue has intensified due to both geographical factors and the wider impacts of global warming, making it a critical concern in poultry production (6, 7). The thermoneutral zone (TNZ) refers to the range of environmental temperatures in which broilers can maintain a balance between evaporative heat loss and metabolic heat production, ensuring their comfort and health (8). When temperatures exceed this zone, the welfare of broilers can deteriorate significantly. In tropical and subtropical regions, the combination of high relative humidity and elevated temperatures creates substantial challenges for effective broiler management (3). Increased temperatures and humidity can lead to heat stress, adversely affecting the growth performance and overall efficiency of broiler chickens (9, 43).

Although broilers in temperate climates typically thrive in intensive farming systems with controlled microclimatic conditions, this is not always true in less developed regions, where broiler farming depends on natural ventilation and open-sided housing (10). These conditions make broilers more susceptible to heat stress (11). Cloacal temperature (CT) is a valuable physiological marker for assessing heat stress, reflecting the core body temperature of the birds (12). Meanwhile, body surface temperature (BST) provides insight into how effectively broilers manage heat dissipation through mechanisms like vasodilation, which helps to release heat through the body’s surface (13). To combat the detrimental effects of thermal stress, dietary interventions can play a critical role (8, 14). Supplements with anti-stress and antioxidant properties, such as probiotics and ascorbic acid, have shown promise in enhancing broiler productivity and resilience (6, 15, 16).

Probiotics are microorganisms that have the ability to fight certain pathogens within the gastrointestinal tract of chicken (17–19, 41). They are generally given as feed additives in sufficient amounts and highly beneficial effects have been noticed in the field (20). Certain bacterial and fungal species have presented promising results as efficient probiotics in both animals and chicken (21, 22). Yeasts like *Saccharomyces cerevisiae* are highly beneficial in stabilizing the gut microbiota along with reducing the risk of disease occurrence (23, 24). Probiotics, such as *Saccharomyces cerevisiae* play a crucial role in alleviating heat stress in broiler chickens through several mechanisms (25). Ascorbic acid, widely recognized as vitamin C, is a powerful antioxidant that plays a crucial role in protecting cells from oxidative stress caused by free radicals. Its potential benefits in managing heat stress, particularly in livestock and poultry, are well-documented (26). While probiotics and ascorbic acid offer distinct benefits, their effectiveness as anti-stress agents may vary based on dosage, administration method, and the specific conditions of the poultry environment (27–30). Their impact on the hypothalamic–pituitary–adrenal axis has made them an effective agent for combating stress, leading to improved resilience and overall well-being by modulating the body’s stress response mechanisms. This study examined the effectiveness of probiotics (*Saccharomyces cerevisiae*) and ascorbic acid as agents to alleviate stress in broiler chickens. It utilized CT and BST as biomarkers for heat stress to assess their impact. To our knowledge, no research has evaluated the combined effects of the probiotic *Saccharomyces cerevisiae* and ascorbic acid in mitigating heat stress impacts on broiler chickens at the time this study was conducted. The objective of this study was to assess the anti-stress effects of both the probiotic and ascorbic acid in broiler chickens during the challenging summer months, using CT and BST as biomarkers for heat stress.

## 2 Materials and methods

### 2.1 Environmental conditions in the experimental sites

After the brooding period, the chickens were exposed to the challenging thermal conditions typical of the hot summer season in Pretoria, South Africa. These conditions were characterized by high relative humidity, exceeding 65–70%, and elevated ambient temperatures exceeding 18–26°C, which induced heat stress in chickens raised in tropical climates (6).

### 2.2 Experimental animals and management

Ceramic heaters set to 34°C were employed to provide the necessary warmth during the brooding period for the broiler chicks, which lasted 14 days. To maintain biosecurity, footbaths containing F10 Super Concentrate (Health and Hygiene (Pty) Ltd., Roodepoort, South Africa) at a dilution of 1:500 were provided. Additionally, all personnel were required to use designated footwear and clothing. Fifty-six chickens were used in this study and divided into four groups of 14. Group I served as the control, Group II received the probiotic, Group III was given ascorbic acid, and Group IV received both the probiotic and ascorbic acid. Probiotics and ascorbic acid were incorporated into the chickens’ feed from D1 to D35. They were administered at a dose of 1 g/kg of feed (31) and 200 mg/kg of feed (15), respectively both singly and in combination. Each broiler chicken was individually marked with color-coded markings and wing tags to ensure precise record-keeping.

### 2.3 Experimental measurements

#### 2.3.1 Thermal environmental parameters

An electronic sensor (Hobo) was installed in the poultry pen to continuously monitor ambient temperature (AT) and relative humidity (RH). The chickens were brooded for 2 weeks at 34°C as earlier mentioned, after which they were exposed to the naturally occurring ambient conditions. On D21, D28, and D35 of the experiment, AT and RH measurements were recorded twice daily to capture the diurnal variations. The temperature-humidity index (THI) was calculated using the following formula:

THI = (1.8 × AT + 32) – (0.55–0.55 × RH) × [(1.8 × AT + 32)–58]

where THI = temperature-humidity index, AT = Ambient temperature (°C) and RH = Relative humidity (%) (6).

#### 2.3.2 Cloacal and body surface temperature measurements

A digital clinical thermometer (Zhengzhou AiQURA Intelligent Technology Co., Ltd., China) was used to record CT on D21, D28 and D35 of the study. These CT measurements were taken concurrently with recordings of AT and RH. For BST measurements, seven broiler chickens from each group were randomly selected on D21, D28 and D35 of the study. Body surface temperature was assessed using an infrared thermometer (Rutland Industries, South Africa).

#### 2.3.3 Calculation of convective and conductive heat loss

Sensible heat loss by convection and conduction to the environment in broiler chickens was calculated using a modified formula (32):

Q_c_ = A_s_ × h (T_s_ − T_at_)

Where:

Q_c_ is conductive and convective heat loss;

A_s_ is the surface area of the bird (m^2^) (A_s_ = 3.86 × MC^0.74^);

MC is the body mass of the broiler chicken (kg);

hc is the heat transfer coefficient (hc = 0.336 × 4.184 × (1.46 + √V_AR_ × 100));

V_AR_ is air velocity (V_AR_ = 0);

T_s_ is the average surface temperature of birds (°C) and

T_at_ is the ambient temperature (°C).

### 2.4 Statistical analysis

The data were log transformed to achieve a normal distribution, which is essential for the validity of subsequent analyses. After the data were normalized, they underwent repeated measures analysis of variance (ANOVA), to determine differences between the means of the control and treatment groups. Tukey’s HSD test was employed, with significance set at 0.05. The analysis was performed using SPSS Statistics for Windows, Version 27 (Armonk, NY: IBM Corp).

## 3 Results

### 3.1 Ambient temperature and cloacal temperature responses

On D21, D28, and D35 of the study period, AT values exceeded the thermoneutral zone recommended for chickens (Table 1). On D21, the CT in the probiotic group was significantly lower (40.84 ± 0.05; *p* < 0.05) at 19:00 h which was the last reading for the day when compared to the control group (41.69 ± 0.18). Additionally, at 7:00 h (which was the start of the procedure) of D35, the CT values recorded in the probiotic, ascorbic acid and the co-administered groups were significantly lower (40.36 ± 0.18; 40.97 ± 0.15 and 41.01 ± 0.16, respectively; *p* < 0.05) than those for the control group (Table 2).

**Table 1:** Temperature and humidity indices on days 21, 28, and 35 of the study.

Time (h)	DBT (°C)	RH (%)	THI
7:00	27.67 ± 0.33 (27–28)	83.33 ± 2.19 (79–86)	27.33 ± 0.32 (26.7–27.7)
9:00	28.33 ± 0.33 (28–29)	74.67 ± 2.19 (72–73)	27.80 ± 0.31 (27.4–28.4)
11:00	28.67 ± 0.33 (28–29)	79.00 ± 0.00 (79)	28.27 ± 0.33 (27.6–28.6)
13:00	33.33 ± 1.67 (30–35)	81.67 ± 4.33 (73–86)	32.80 ± 1.70 (29.4–34.6)
15:00	34.00 ± 1.00 (33–36)	84.00 ± 2.00 (80–86)	33.40 ± 1.00 (32.4–35.4)
17:00	31.33 ± 0.67 (30–32)	80.00 ± 0.00 (80)	30.87 ± 0.64 (29.6–31.6)
19:00	28.33 ± 0.33 (28–29)	77.00 ± 2.00 (73–79)	27.87 ± 0.27 (27.6–28.4)
Overall mean ± SEM	30.24 ± 0.60 (27–36)	79.95 ± 1.00 (72–86)	29.76 ± 0.59 (26.7–35.4)

RH, Relative humidity; DBT, Dry-bulb temperature; THI, Temperature-humidity index.

**Table 2:** Changes in cloacal temperature of broiler chickens given probiotic and ascorbic acid.

Day	Time (h)	Group			
		Control	Probiotic	Ascorbic acid	Probiotic + AA
21	07:00	41.21 ± 0.27^a^	39.98 ± 0.13^b^	40.78 ± 0.11^a^	41.04 ± 0.15^a^
	09:00	40.35 ± 0.30^a^	40.07 ± 0.20^a^	40.46 ± 0.11^a^	40.96 ± 0.08^b^
	11:00	40.99 ± 0.25^a^	40.72 ± 0.62^a^	40.85 ± 0.09^a^	41.06 ± 0.09^a^
	13:00	41.00 ± 0.25^a^	40.45 ± 0.08^a^	40.80 ± 0.11^a^	40.83 ± 0.12^a^
	15:00	41.14 ± 0.20^a^	40.84 ± 0.06^a^	40.97 ± 0.04^a^	40.98 ± 0.09^a^
	17:00	41.53 ± 0.15^a^	40.36 ± 0.10^b^	41.11 ± 0.12^a^	41.02 ± 0.08^a^
	19:00	41.69 ± 0.18^a^	40.84 ± 0.05^b^	41.36 ± 0.14^a^	41.20 ± 0.08^a^
28	07:00	41.63 ± 0.15^a^	39.80 ± 0.18^c^	40.63 ± 0.14^b^	40.52 ± 0.16^b^
	09:00	41.39 ± 0.32^a^	40.07 ± 0.20^b^	40.46 ± 0.12^b^	40.96 ± 0.08^a^
	11:00	41.79 ± 0.22^a^	40.78 ± 0.22^b^	41.03 ± 0.22^b^	41.01 ± 0.13^b^
	13:00	41.69 ± 0.11^a^	40.68 ± 0.13^b^	40.78 ± 0.13^b^	40.75 ± 0.12^b^
	15:00	41.14 ± 0.20^a^	40.84 ± 0.06^a^	40.97 ± 0.06^a^	40.98 ± 0.09^a^
	17:00	41.71 ± 0.14^a^	40.87 ± 0.16^b^	41.03 ± 0.16^b^	40.86 ± 0.14^b^
	19:00	41.69 ± 0.17^a^	41.10 ± 0.14^a^	41.23 ± 0.14^a^	41.07 ± 0.09^a^
35	07:00	41.53 ± 0.20^a^	40.36 ± 0.18^b^	40.97 ± 0.15^a^	41.01 ± 0.16^a^
	09:00	41.15 ± 0.27^a^	40.16 ± 0.18^b^	40.49 ± 0.12^b^	40.90 ± 0.14^a^
	11:00	41.24 ± 0.24^a^	40.86 ± 0.12^a^	40.81 ± 0.11^a^	41.02 ± 0.13^a^
	13:00	41.47 ± 0.22^a^	40.59 ± 0.13^b^	40.89 ± 0.14^b^	40.73 ± 0.12^b^
	15:00	41.24 ± 0.20^a^	40.81 ± 0.12^a^	40.98 ± 0.11^a^	40.86 ± 0.11^a^
	17:00	41.49 ± 0.16^a^	40.34 ± 0.12^b^	41.09 ± 0.12^a^	40.94 ± 0.11^a^
	19:00	41.66 ± 0.17^a^	40.94 ± 0.12^b^	41.27 ± 0.13^a^	40.98 ± 0.12^b^

Mean values with different superscript letters along the same row are significantly different at *p* < 0.05. *n* = 14.

### 3.2 Body surface temperature (BST)

On D21, D28 and D35, broiler chickens in the probiotic, ascorbic acid and the co-administered groups exhibited significantly higher (*p* < 0.05) temperatures in the comb and wing due to heat dissipation to the surrounding. There was a significantly lower (*p* < 0.05) temperature in the foot, back and head of the broiler chickens in the treatment group in comparison with the control during the study (Table 3).

**Table 3:** Variations in head, comb, wing, back and foot temperature of broiler chickens given probiotic and ascorbic acid.

Area	Day	Time (h)	Control	Treatment groups		
				Probiotic	Ascorbic acid	Probiotic + Ascorbic acid
Head	21	07:00	37.01 ± 0.53^a^	36.79 ± 0.17^a^	35.87 ± 0.15^a^	33.00 ± 1.38^b^
		13:00	36.03 ± 0.23^a^	37.06 ± 0.23^a^	34.00 ± 0.85^b^	34.57 ± 0.91^b^
		19:00	36.34 ± 0.18^a^	37.16 ± 0.28^a^	35.69 ± 0.48^b^	32.34 ± 0.68^c^
	28	07:00	37.10 ± 0.43^a^	36.26 ± 0.19^a^	35.83 ± 0.21^a^	32.59 ± 1.49^b^
		13:00	35.91 ± 0.25^a^	36.89 ± 0.25^a^	35.09 ± 0.23^a^	35.07 ± 0.73^a^
		19:00	37.89 ± 0.62^a^	37.00 ± 0.21^a^	38.20 ± 0.60^a^	36.06 ± 0.41^b^
	35	07:00	37.17 ± 0.42^a^	36.67 ± 0.17^a^	35.93 ± 0.22^a^	36.16 ± 0.86^a^
		13:00	36.79 ± 0.49^a^	36.91 ± 0.28^a^	36.17 ± 0.32^a^	35.97 ± 0.51^a^
		19:00	37.49 ± 0.41^a^	35.84 ± 0.50^b^	35.69 ± 0.65^b^	35.20 ± 0.29^b^
Comb	21	07:00	36.63 ± 0.42^a^	35.97 ± 0.20^a^	35.51 ± 0.11^a^	35.21 ± 1.51^a^
		13:00	35.86 ± 0.16^a^	36.21 ± 0.07^a^	33.19 ± 0.58^b^	33.50 ± 0.81^b^
		19:00	36.96 ± 0.25^a^	36.53 ± 0.10^a^	37.73 ± 0.44^a^	36.00 ± 0.69^a^
	28	07:00	36.37 ± 0.36^a^	36.76 ± 0.48^a^	35.59 ± 0.18^a^	36.30 ± 0.17^a^
		13:00	35.74 ± 0.18^a^	36.24 ± 0.21^a^	35.19 ± 0.50^a^	35.64 ± 0.38^a^
		19:00	35.60 ± 0.47^a^	36.41 ± 0.11^a^	37.73 ± 0.48^b^	36.14 ± 0.48^a^
	35	07:00	35.27 ± 0.42^a^	37.59 ± 0.58^b^	36.23 ± 0.26^a^	37.14 ± 0.41^b^
		13:00	34.53 ± 0.49^a^	36.50 ± 0.33^b^	35.90 ± 0.23^a^	36.43 ± 0.41^b^
		19:00	35.93 ± 0.42^a^	37.96 ± 0.67^b^	37.53 ± 0.41^b^	36.57 ± 0.54^a^
Wing	21	07:00	36.23 ± 0.34^a^	39.16 ± 0.27^b^	40.47 ± 0.32^c^	37.23 ± 0.50^a^
		13:00	39.83 ± 0.20^a^	39.23 ± 0.46^a^	39.01 ± 0.50^a^	38.44 ± 0.33^a^
		19:00	39.83 ± 0.20^a^	40.07 ± 0.13^b^	39.90 ± 0.35^a^	38.30 ± 0.07^a^
	28	07:00	35.76 ± 0.16^a^	39.07 ± 0.29^c^	40.09 ± 0.26^c^	37.39 ± 0.48^b^
		13:00	39.83 ± 0.48^a^	40.54 ± 0.40^a^	39.30 ± 0.72^a^	40.44 ± 0.47^a^
		19:00	37.87 ± 0.84^a^	40.34 ± 0.26^b^	40.59 ± 0.34^b^	39.73 ± 0.68^b^
	35	07:00	35.71 ± 0.36^a^	39.30 ± 0.30^c^	40.40 ± 0.28^c^	37.53 ± 0.43^b^
		13:00	36.81 ± 0.73^a^	39.14 ± 0.45^b^	38.90 ± 0.49^b^	37.87 ± 0.65^b^
		19:00	37.81 ± 1.09^a^	40.10 ± 0.13^b^	39.71 ± 0.31^b^	38.86 ± 0.30^b^
Back	21	07:00	37.43 ± 0.59^a^	36.06 ± 0.23^a^	36.96 ± 0.38^a^	31.81 ± 0.84^b^
		13:00	36.36 ± 0.11^a^	36.61 ± 0.28^a^	36.69 ± 0.91^a^	34.89 ± 0.62^a^
		19:00	37.36 ± 0.11^a^	34.26 ± 0.33^c^	36.66 ± 0.88^b^	35.50 ± 0.69^b^
	28	07:00	36.87 ± 0.78^a^	35.83 ± 0.25^a^	36.67 ± 0.45^a^	35.61 ± 0.68^a^
		13:00	36.37 ± 0.13^a^	36.53 ± 0.18^a^	37.27 ± 0.85^a^	35.57 ± 0.36^a^
		19:00	39.96 ± 0.97^a^	37.16 ± 0.35^b^	38.69 ± 0.95^a^	36.07 ± 0.37^c^
	35	07:00	37.36 ± 0.67^a^	36.01 ± 0.26^a^	36.89 ± 0.38^a^	36.39 ± 0.88^a^
		13:00	37.56 ± 0.62^a^	36.04 ± 0.30^b^	36.06 ± 0.81^b^	35.60 ± 0.40^b^
		19:00	38.31 ± 0.13^a^	36.04 ± 0.36^b^	37.46 ± 0.82^b^	35.64 ± 0.21^c^
Foot	21	07:00	36.74 ± 0.57^a^	35.94 ± 0.18^a^	36.34 ± 0.25^a^	36.06 ± 0.19^a^
		13:00	35.39 ± 0.17^a^	35.50 ± 0.24^a^	34.16 ± 1.04^a^	35.34 ± 1.14^a^
		19:00	38.30 ± 0.16^a^	37.57 ± 0.36^a^	37.33 ± 0.19^a^	35.09 ± 1.60^b^
	28	07:00	36.70 ± 0.51^a^	35.89 ± 0.18^a^	36.24 ± 0.24^a^	35.94 ± 0.22^a^
		13:00	35.50 ± 0.20^a^	35.47 ± 0.21^a^	34.01 ± 0.98^b^	34.36 ± 0.95^b^
		19:00	39.16 ± 1.02^a^	37.57 ± 0.36^b^	38.33 ± 0.19^a^	35.66 ± 1.27^c^
	35	07:00	36.67 ± 0.59^a^	35.86 ± 0.19^b^	36.27 ± 0.23^a^	36.03 ± 0.25^a^
		13:00	35.71 ± 0.22^a^	35.26 ± 0.29^a^	34.41 ± 0.83^b^	36.06 ± 0.80^a^
		19:00	37.50 ± 0.66^a^	37.29 ± 0.36^a^	37.59 ± 0.59^a^	36.46 ± 1.08^a^

Mean values with different superscript letters along the same row are significantly different at *p* < 0.05. *n* = 14.

### 3.3 Convective and conductive heat loss

**Figure 1:**
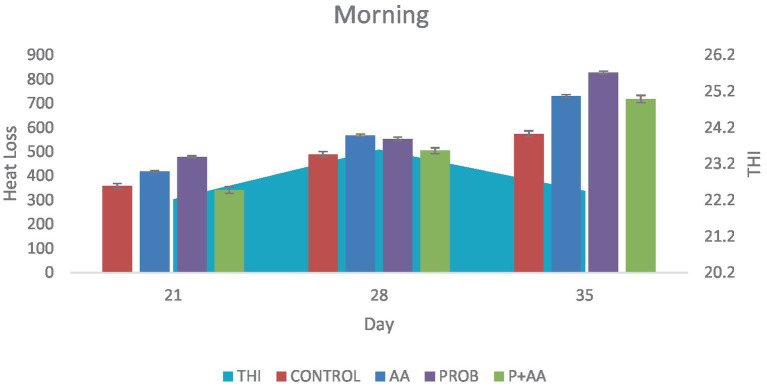
Convective and conductive heat loss obtained during the morning hours of the study period. The THI was within the TNZ stipulated for broiler chickens which influenced the degree of heat loss positively during this period of the study (*n* = 7). THI; temperature-humidity index, TNZ; thermoneutral zone.

**Figure 2:**
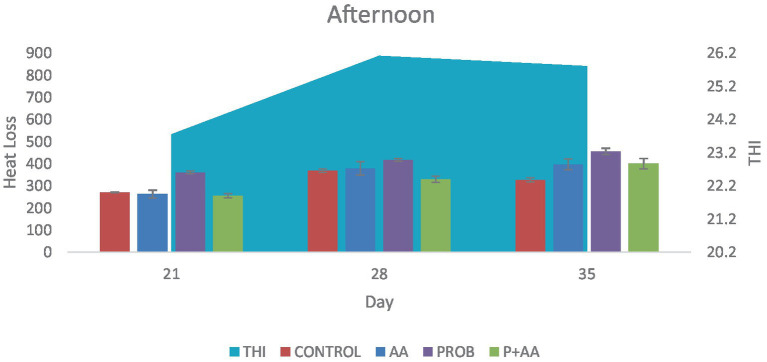
Convective and conductive heat loss obtained during the afternoon hours of the study period. The THI exceeded the TNZ for broiler chickens which influenced the degree of heat loss negatively during this period of the study (*n* = 7). THI, temperature-humidity index; TNZ, thermoneutral zone.

**Figure 3:**
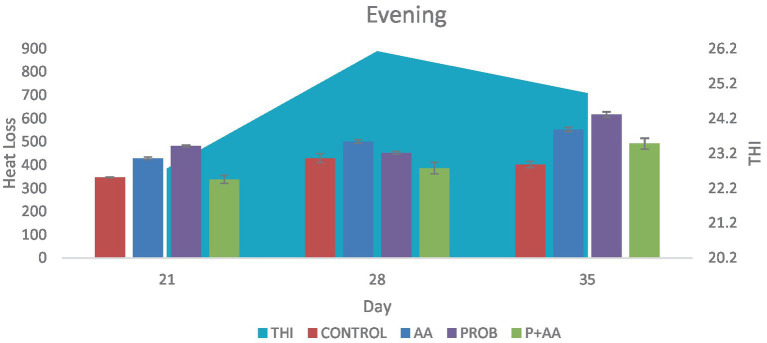
Convective and conductive heat loss obtained during the evening hours of the study period. The probiotic and ascorbic acid groups had a significantly higher (*p* < 0.05) value of heat loss when compared with the control group on D21 and D35. The THI was outside the TNZ stipulated for broiler chickens which negatively influenced the degree of heat loss during this period of the study (*n* = 7). THI, temperature-humidity index; TNZ, thermoneutral zone.

**Figure 4:**
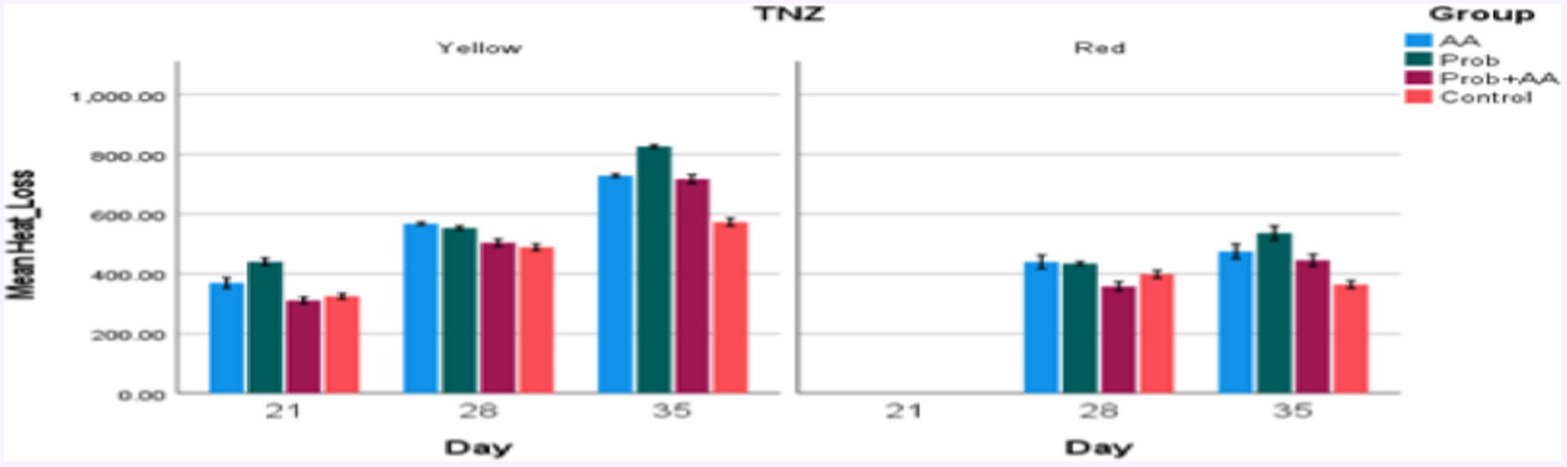
Convective and conductive heat loss within (yellow zone) and outside (red zone) the TNZ in broiler chickens treated with probiotic and ascorbic acid. At D21, heat loss values obtained were within the TNZ, while those recorded on D28 and D35 surpassed the TNZ stipulated for broiler chickens during the afternoon and evening periods of the study (*n* = 7).

On D35 heat loss recorded in the treatment groups was significantly higher (*p* < 0.05) compared to the control group. During the morning period of the study, the THI remained within the thermoneutral zone (TNZ) (Figure 1). However, at noon and in the evening, although the THI exceeded the TNZ, there was a significant difference (*p* < 0.05) in heat loss between the probiotic group and the control group (Figures 2, 3). On D21, all recorded heat loss values were within the TNZ, indicated by the yellow zone, for broiler chickens. However, on days 28 and 35 of the study, the heat loss values exceeded the TNZ, falling into the red zone (Figure 4).

## 4 Discussion

The elevated CT values observed in the control group suggest that these birds experienced a decreased ability to cope with thermal stress as they aged. The lack of any intervention in this group likely contributed to the higher CT, especially noticeable during the afternoon and evening hours. This observation is consistent with Egbuniwe et al. (26), who reported increased CT in chickens deprived of betaine and ascorbic acid, indicating that such deficiencies impair the birds’ thermal regulation. In contrast, the probiotic-treated group exhibited significantly lower CT values, which can be attributed to the anti-stress properties of probiotic through its influence on the HPA axis. Yeast probiotics (*Saccharomyces cerevisiae*) have been shown to be effective anti-stress agents, improving broiler performance and heat tolerance when administered in appropriate doses during periods of thermal stress (6, 25). This finding supports Sugiharto et al. (33), who demonstrated that probiotics could modulate the adverse effects of increased metabolic heat production associated with higher body weight gains, thus enhancing heat dissipation in broilers. The lack of an additive effect on CT in the group receiving both probiotics and ascorbic acid may suggest that the mechanisms through which these two treatments operate are overlapping or synergistic in a way that does not result in enhanced benefits when combined during this study. Additionally, the physiological responses of the chickens to heat stress might have reached a maximum threshold, preventing any additional effects from the combined treatment. Despite ascorbic acid’s role in reducing corticosterone levels through a negative feedback mechanism (15, 34), it did not demonstrate superior efficacy compared to the probiotic alone in mitigating thermal stress. This outcome suggests that while ascorbic acid can contribute to stress reduction, its impact may be limited by factors such as the specific dose used, the bioavailability of the antioxidant, or the inherent variability in the susceptibility of broiler chickens to these treatments. Additionally, it is worth considering that the efficacy of antioxidants can be influenced by their interaction with other components of the diet and environmental conditions (35). The varying responses observed in this study highlight the need for further research to optimize the use of these agents and understand their mechanisms in managing heat stress.

The lower temperatures recorded in the head, back, and feet of the treatment groups suggest that the probiotic and ascorbic acid played a significant role in enhancing the birds’ ability to manage heat stress. During periods of heat stress, broiler chickens typically increase their oxygen intake to support thermoregulatory mechanisms such as evaporative cooling through panting (8, 36). This heightened oxygen consumption can lead to the accumulation of reactive oxygen species (ROS), which are byproducts of oxygen metabolism. When endogenous antioxidants are insufficient to counteract these ROS, oxidative stress can occur. The inclusion of exogenous antioxidants, such as *Saccharomyces cerevisiae* and ascorbic acid, may help neutralize these ROS, thereby reducing oxidative stress and supporting better thermal regulation (6, 15). The increased BST observed in the control group likely reflects the chickens’ impaired ability to regulate heat, as indicated by their elevated CT. This supports the findings of Kim et al. (37), who noted that BST is a sensitive indicator of heat stress levels, with higher environmental temperatures leading to its increase. Although the study focused on laying hens, the relationship between environmental temperature, heat stress, and BST is also applicable to broiler chickens. The findings indicated that while the probiotic may help alleviate stress, combining it with ascorbic acid did not yield an additive effect on core temperature. This underscores the need for targeted approaches in broiler management. To optimize poultry welfare and performance during warmer months, practical recommendations for broiler producers are essential. These include incorporating probiotics into feeding regimens, monitoring environmental conditions, ensuring proper hydration, and adjusting brooding practices. By implementing these strategies, producers can enhance the resilience of their flocks and improve overall production outcomes in the face of climate-related challenges.

In the morning hours of the study, the THI was within the TNZ ideal for optimal broiler production. This favorable THI allowed for effective thermoregulation through convective and conductive heat loss, which was evident in the treatment groups as THI is the descriptive indicator of heat stress [(38); Xinyao et al., 2022]. Throughout the study, broiler chickens accumulated heat from both environmental sources and metabolic processes. However, the primary focus was on assessing sensible heat loss in the treatment and control groups. It is important to acknowledge that while the antioxidants contributed to increased heat loss, the overall effectiveness of heat dissipation was also influenced by the THI. Variations in THI during different periods of the study likely impacted the heat loss dynamics. Tao and Xin (39), found that the optimal THI for broiler production is around 21, suggesting that maintaining THI within this range is crucial for minimizing heat stress and ensuring optimal performance. This implies that alongside antioxidant supplementation, managing THI effectively is essential for enhancing broiler welfare and productivity. During the afternoon and evening hours of the study, the THI exceeded the TNZ optimal for broiler chickens’ production. Such conditions are expected to reduce heat loss through conduction and convection. Despite this, the probiotic group demonstrated a higher degree of heat loss compared to the control group on D21 and D35. This increased heat loss in the probiotic group can be attributed to the antistress effect of this agent. These findings align with the research of Sinkalu et al. (40) and Aluwong et al. (6), who both identified that THI levels above 21 induce heat stress in broiler chickens. Their studies utilized CT as a biomarker to assess heat stress, corroborating the observation that high THI contributes to increased heat stress.

The TNZ is the optimal temperature range in which broiler chickens can maintain their physiological functions without needing to expend extra energy for thermoregulation (8). This zone, also known as the comfort zone, is crucial for achieving peak performance and welfare in broiler chickens (13). Within this range, known as the zone of comfort (yellow zone), the broilers can effectively manage their body temperature and perform optimally. However, when ambient temperatures exceed this range, entering the zone of discomfort (red zone), the chickens experience increased stress and reduced performance (39). During the study, THI values recorded on D21 and in the morning hours of D28 and D35 remained within the TNZ. These favorable conditions facilitated effective thermoregulation in the broiler chickens, as evidenced by efficient heat loss through convection and conduction. The ability to maintain normal physiological functions and comfort levels was thus supported. In contrast, the THI values recorded during the afternoon and evening hours of D28 and D35 were above the TNZ, which significantly impaired the chickens’ ability to regulate their body temperature. This was reflected in the reduced effectiveness of heat dissipation mechanisms, leading to compromised welfare and performance (26). The elevated temperatures in these periods resulted in increased physiological stress and diminished comfort for the broilers. Our study indicates that higher AT beyond the TNZ negatively affects the thermoregulatory processes in broiler chickens, particularly when no anti-stress interventions are applied. The data suggest that as AT increased and exceeded the TNZ, the capacity for effective thermoregulation diminishes, highlighting the critical need for environmental management and stress mitigation strategies. This reinforces the importance of maintaining environmental conditions within the TNZ to optimize broiler health and productivity (10, 44). Additionally, the findings underscore the potential benefits of implementing anti-stress measures, such as dietary supplements like probiotics (*Saccharomyces cerevisiae*) and ascorbic acid to support broiler welfare during periods of thermal stress. Further research should explore the interaction between various antioxidant types and environmental conditions to develop comprehensive strategies for managing heat stress in broiler production. This could include optimizing antioxidant dosages and combining them with environmental controls to achieve the best outcomes for broiler health and performance. Understanding how fluctuations in THI influence heat stress and performance at different growth phases can inform more targeted management practices. Also, the effects of different combinations and dosages of antioxidants, including probiotics and ascorbic acid should be studied, to determine their synergistic potential in mitigating heat stress.

## 5 Conclusion

The study highlights the importance of maintaining ambient temperatures within the TNZ to optimize the welfare and performance of broiler chickens. It found that when THI values are within the TNZ, chickens effectively regulate their body temperature, but exceedance leads to impaired thermoregulation, elevated CT, and increased heat stress. Practical recommendations for producers include supplementing feed with probiotics, which have been effective in reducing heat stress effects, as opposed to ascorbic acid, which has a less pronounced impact during this study. Producers should, therefore, integrate probiotics into their feeding strategies, especially during warmer months. Future research should aim to optimize dosages and combinations of probiotics and ascorbic acid, explore their specific mechanisms, and assess long-term effects on health and productivity. Overall, maintaining temperatures within the TNZ and implementing these anti-stress interventions can enhance broiler resilience and improve production outcomes.

## Data Availability

The raw data supporting the conclusions of this article will be made available by the authors, without undue reservation.
